# The Renewed Role of Sweep Functions in Noisy Shortcuts to Adiabaticity

**DOI:** 10.3390/e23070897

**Published:** 2021-07-14

**Authors:** Michele Delvecchio, Francesco Petiziol, Sandro Wimberger

**Affiliations:** 1 Department of Mathematical, Physical and Computer Sciences, University of Parma, Parco Area delle Scienze 7/A, 43124 Parma, Italy; 2 National Institute for Nuclear Physics (INFN), Milano Bicocca Section, Parma Group, Parco Area delle Scienze 7/A, 43124 Parma, Italy; 3 Institut für Theoretische Physik, Technische Universität Berlin, Hardenbergstr. 36, 10623 Berlin, Germany

**Keywords:** adiabatic evolution, open quantum systems, quantum control

## Abstract

We study the robustness of different sweep protocols for accelerated adiabaticity following in the presence of static errors and of dissipative and dephasing phenomena. While in the noise-free case, counterdiabatic driving is, by definition, insensitive to the form of the original sweep function, this property may be lost when the quantum system is open. We indeed observe that, according to the decay and dephasing channels investigated here, the performance of the system becomes highly dependent on the sweep function. Our findings are relevant for the experimental implementation of robust shortcuts-to-adiabaticity techniques for the control of quantum systems.

## 1. Introduction

One of the most successful methods for quantum control is given by the adiabatic passage [1,2]. The adiabatic passage has its root in the adiabatic theorem of quantum mechanics [3], which states that if a system parameter is driven in a sufficiently slow manner in time, resulting in a time-dependent Hamiltonian $H_{0}\left(t\right)$, then the populations of the instantaneous eigenstates of $H_{0}\left(t\right)$ are preserved. A system initially prepared, for instance, in the ground state of $H_{0}\left(t_{\mathrm{in}}\right)$ is driven by the adiabatic evolution toward the ground state of $H_{0}\left(t_{\mathrm{fin}}\right)$, which is the desired state to be prepared assuming that no level-crossing occurs. Unless the evolution takes place in infinite time, errors due to the finite duration of the protocol accumulate during the evolution. These errors can be reduced by optimizing the rate at which the parameters are varied, i.e., by designing appropriate *sweep functions* [4,5]. Studies on the performance of some specific sweep functions in the presence of noise have been performed, for instance, in the context of adiabatic quantum computing [6,7].

However, in real applications, adiabatic dynamics, which is slow by definition, becomes often impractical because it might require evolution times that are longer than the coherence time of the system. This has motivated the development of the so-called shortcuts to adiabaticity (STA), namely of a variety of techniques for achieving adiabatic evolution though in a shorter time [8,9,10]. For instance, this can be achieved by requiring that the controlled dynamics match the adiabatic one only at the beginning and at the end of the protocol [11,12,13,14,15]. In general, STA protocols accelerate an adiabatic sweep through the introduction of additional control fields. These fields are designed in order to exactly counteract nonadiabatic transitions throughout the evolution, resulting in an exact following of the instantaneous eigenvectors of the driven Hamiltonian $H_{0}\left(t\right)$. The trade-off for this perfect adiabatic following is that the extra fields are typically very hard to be realized in practice [8,11,16,17,18,19,20]. This longstanding problem in the field has attracted much attention and has been tackled from different angles by resorting, for instance, to Floquet engineering methods [16,21,22,23,24,25,26,27,28,29].

In this work, we take a different perspective: we take the possibility to realize the additional fields for granted, and we rather investigate robustness properties of the STA. The specific question that we address is the following. Provided that $H_{0}\left(t\right)$ adiabatically always connects the initial and target quantum states, for any choice of the sweep function in $H_{0}\left(t\right)$, one can construct the corresponding STA fields. In this way, one can obtain a protocol that gives the desired state preparation with unit fidelity. At the level of the isolated and error-free system, there apparently is no preferential choice for the specific time-dependence in $H_{0}\left(t\right)$. Our goal, instead, is to investigate whether this apparent equivalence of different protocols based on different sweep functions breaks down when control imperfections or noise is introduced into the system. A similar problem was addressed in [30], though for a specific scenario different from ours, where the authors optimized the control pulse for counteracting the effect of the specific noise in an experimental setup. Moreover, in an open system environment, in ref. [31], the authors investigated the performance of a population transfer in the presence of collisional dephasing, using the counterdiabatic protocol for three and four level systems. Other related studies can be found in [32,33], where the authors explored the effect of dephasing in one adiabatic protocol and used a different STA technique based on dynamical invariants. We, instead, perform a systematic study of three different sweep functions in the presence of static errors and dissipative and dephasing effects, with counterdiabatic driving as the STA method. We indeed find out that, while for imperfections in the energy gap, the three sweep functions are all very robust, in the presence of dephasing or decay, one may choose the optimal sweep function, according to the type of noise affecting the system.

This paper is organized as follows: we first present in Section 2 well-known material on adiabatic and counterdiabatic driving, as well as the Lindblad master equation used for studying the open quantum systems in the following. We then analyze in Section 3.1 the fundamental problem of driving a two-level system through an avoided crossing in the energy spectrum for the purpose of realizing a population inversion. In this context, we analyze in the first place the performance of different classes of sweep functions, and the corresponding STA protocol when imperfections in the system parameters are introduced. Then, robustness against dissipative and dephasing effects is analyzed by studying the open system dynamics produced by different coupling schemes of the two-level system with the environment. To go beyond the prototypical two-state model, Section 3.2 extends the analysis to a protocol for generating entanglement between two qubits, by investigating the effect of local qubit dissipation and decoherence on a sample of STA schemes. Finally, Section 4 sums up our findings.

## 2. Theoretical Background

In this section, we briefly review first the idea of adiabatic driving in Section 2.1. Then, in Section 2.2, we introduce counterdiabatic driving, which is known as one of the first shortcuts-to-adiabaticity techniques, and finally in Section 2.3, the Lindblad master equation for the description of the open quantum systems.

### 2.1. Adiabatic Sweep Functions

We analyze control protocols that can be generically described through a Hamiltonian of the following form:

(1) $${\hat{H}}_{0}\left(t\right)=f\left(t\right){\hat{H}}_{1}+{\hat{H}}_{2}$$

where f(t) is the sweep function describing the slow parameter variation, which realizes the adiabatic passage. Depending on the specific problem at hand, the specific choice of f(t) can mitigate the effects of some particular error sources. We choose the following sweep functions: (i) *linear* (Landau–Zener), (ii) *polynomial* and (iii) *Roland–Cerf*. While the *linear* one (i) is derived from the classical Landau–Zener problem, the other two are found by following two kinds of approaches: boundary cancellation methods (BCMs) [5] for (ii) and local adiabatic drivings (LADs) [4,34] for (iii). BCM are constructed by noting that higher-order perturbative expansions of the nonadibatic errors depend on higher-order time derivatives of the Hamiltonian at the beginning and at the end of the protocol. Such errors can thus be reduced by choosing sweep functions with vanishing time derivatives at those points. Conversely, LAD keep such errors under control by discretizing the evolution in small time steps and adapting the sweep rate such that a time-local adiabaticity condition holds in any such time step. In particular, we designed our polynomial function by setting to zero the first two derivatives at the beginning and at the end of the evolution as the BCMs required. The Roland–Cerf function is derived by imposing the local adiabatic condition as described by the LADs method (see Appendix A). According to the target we wanted to obtain, the sweep functions were consequently adapted. For the one qubit case, we considered ${\hat{H}}_{1}=\alpha {\hat{\sigma }}_{z}$, ${\hat{H}}_{2}=g{\hat{\sigma }}_{x}$, where α>0 is the sweep parameter, *g* the coupling constant and ${\hat{\sigma }}_{i}\left(i=x,y,z\right)$ is the Pauli matrices. Then, we designed the functions to perform a complete population transfer, so that they take the following specific forms:

(2) $$\begin{array}{ccc} \left(i\right) & & f_{L}\left(\tau \right)=f_{0}\left(1-2\tau \right) \end{array}$$

(3) $$\begin{array}{ccc} \left(\mathrm{ii}\right) & & f_{PL}\left(\tau \right)=\frac{f_{0}}{13}\left(13-280\tau^{3}+490\tau^{4}-336\tau^{5}+140\tau^{6}-40\tau^{7}\right) \end{array}$$

(4) $$\begin{array}{ccc} \left(\mathrm{iii}\right) & & f_{RC}\left(\tau \right)=\frac{\left(1-2\tau \right)f_{0}}{\sqrt{1+4\alpha_{g}^{2}f_{0}^{2}\tau \left(1-\tau \right)}}\;, \end{array}$$

where $f\left(0\right)=f_{0}>0$ and τ and $\alpha_{g}$ are, respectively, the time and the sweep parameter in the dimensionless units defined below, before Equation (9). For the two qubits model, instead, the sweep functions are the same as in [35], which are already setup for the realization of an entangling two-qubit gate.

### 2.2. Counterdiabatic Driving (STA)

The type of STA scheme that we consider is the so-called counterdiabatic or transitionless quantum driving as conceived by Berry [36] and Demirplak and Rice [37]. Starting from the system Hamiltonian, we have the following:

(5) $$H_{0}\left(t\right)=\sum_{n}E_{n}\left(t\right)|n(t)\rangle \langle n(t)|\;,$$

where, with instantaneous eigenenergies $E_{n}\left(t\right)$ and eigenvectors |n(t)〉, it is possible to find a Hamiltonian $H\left(t\right)=H_{0}\left(t\right)+H_{\mathrm{CD}}\left(t\right)$ such that, thanks to the correcting term $H_{\mathrm{CD}}\left(t\right)$, the evolution perfectly follows the instantaneous eigenstate |n(t)〉. Such a correcting Hamiltonian is explicitly written as follows:

(6) $$H_{\mathrm{CD}}\left(t\right)=i\hbar \sum_{k\neq n}\sum_{n}\frac{|k(t)\rangle \langle k(t)|\partial_{t}H_{0}\left(t\right)|n(t)\rangle \langle n(t)|}{E_{n}\left(t\right)-E_{k}\left(t\right)}\;.$$

The computation of Equation (6) thus requires the knowledge of all instantaneous eigenstates and eigenvalues of $H_{0}\left(t\right)$ for all times of interest. For the two systems that we consider here, it is possible to compute the corresponding expressions and the two correcting Hamiltonians analytically as showed in the following sections.

### 2.3. Lindblad Master Equation

We study in the following the situation in which the driven system interacts with the surrounding environment, introducing, thus, errors in the control operation. For taking into account dissipative and dephasing effects, we simulate the open dynamics using a Lindblad master equation as follows [38]:

(7) $$\frac{d\rho (t)}{dt}=-\frac{i}{\hbar }\left[H\{f(t)\},\rho (t)\right]+D\left[\hat{c}\right]\rho \left(t\right),$$

where $D\left[\hat{c}\right]\rho \left(t\right)=\hat{c}\rho \left(t\right){\hat{c}}^{†}-\frac{1}{2}{\hat{c}}^{†}\hat{c}\rho \left(t\right)-\frac{1}{2}\rho \left(t\right){\hat{c}}^{†}\hat{c}$ is the Lindblad dissipator. Different types of jump operators $\hat{c}$ are analyzed below, which capture different types of phenomena.

## 3. Results

We first analyze in Section 3.1 the one-qubit system in the presence of static imperfections in the energy gap, and then with different dephasing and decay channels. After that, we extend in Section 3.2 our investigation to two coupled qubits, each independently coupled to the environment.

### 3.1. Two-Level Avoided Crossing

The “simplest non-simple” [39] system for the study of nonadiabatic effects is a two-level system whose energy levels are driven through an avoided crossing. The Hamiltonian of such a system can be written in the following form:

(8) $$H\left(t\right)=\alpha f\left(t\right){\hat{\sigma }}_{z}+g{\hat{\sigma }}_{x}\;.$$

This Hamiltonian physically describes, for instance, two atomic levels driven by a resonant electromagnetic field in a frame rotating at the drive frequency and in the rotating-wave approximation [40]. For g=0, the eigenstates of the system become degenerate when f(t)=0, while for g≠0, they form an avoided crossing of width 2g.

**Figure 1:**
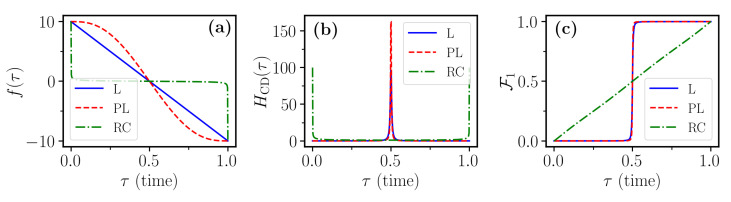
Time dependence, for the one-qubit case, of (**a**) the three sweep functions in Section 2.1; (**b**) the matrix elements of the counterdiabatic term corresponding to the three sweep functions; (**c**) the fidelity $F_{1}$ of Equation (11) for the three sweep functions in the absence of any error and dissipation (γ=0). Parameters used for all the simulations are $T=10,\alpha_{g}=10,f_{0}=10$, in the dimensionless units introduced just before Equation (9).

The temporal profile of the sweep function f(t) controls the evolution speed of the system eigenstates. In Figure 1a, we can distinguish the time dependence of the three sweep functions introduced in Section 2.1. We see that, while the *linear* function maintains the same slope along the entire evolution, the other two functions are designed such that they vary with a non-constant rate. In particular, the *Roland–Cerf* sweep function tends to be faster when the system is far away from the avoided crossing region, and slower nearby it in order to avoid non-adiabatic transitions between the eigenstates. Conversely, the *polynomial* sweep function tends to be constant at the beginning and at the end of the evolution, whereas it is faster than all the others in the vicinity of the avoided crossing.

It is convenient to reparametrize the Hamiltonian of Equation (8) introducing a finite evolution time $t_{f}$ such that the avoided crossing occurs at $t_{f}/2$. Using ℏ=1, we rewrite the parameters in the following natural units $\tau =t/t_{f},T=gt_{f},\alpha_{g}=\alpha /g$ with α>0 so that Equation (8) assumes the following form:

(9) $$H\left(\tau \right)=T\left[\alpha_{g}f\left(\tau \right){\hat{\sigma }}_{z}+{\hat{\sigma }}_{x}\right]\;.$$

**Figure 2:**
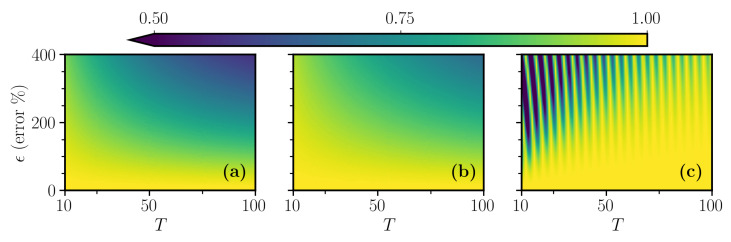
Final fidelity $F_{1}$ of the three sweep functions (**a**) linear, (**b**) polynomial and (**c**) Roland–Cerf, in the presence of the indicated error ϵ. The behavior of (**a**) is very similar to that of (**b**) because the two corresponding control protocols, linear and polynomial, have a very similar fidelity temporal shape, as observed in Figure 1c, while, in the Roland–Cerf protocol, we see characteristic oscillations [35]. These are related to accidental cancellations of higher-order non-adiabatic processes that lead to an accidentally increased performance. The other simulation parameters are the same as in Figure 1.

Now, *T* controls the duration of the evolution and $\alpha_{g}$ is the parameter that controls the initial energy gap between the two eigenstates of the system, for fixed f(0). The counterdiabatic field for the Hamiltonian of Equation (9) can be computed analytically and reads as follows:

(10) $$H_{\mathrm{CD}}\left[f\left(\tau \right)\right]=-\frac{\alpha_{g}\dot{f}\left(\tau \right)}{1+f^{2}\left(\tau \right)}\frac{1}{2}{\hat{\sigma }}_{y}\;,$$

where $\dot{f}\left(\tau \right)$ stands for the time derivative of f(τ). As a first step, the robustness of the counterdiabatic protocols $H_{\mathrm{CD}}\left\{f\left(\tau \right)\right\}$, which we can observe in Figure 1b for the three different sweep functions, is investigated against parameter imperfections. To this end, we introduce a relative error in the parameter $\alpha_{g}$, $\alpha_{g}\to \alpha_{g}\left(1+\epsilon \right)$ while implementing the counterdiabatic protocol computed in the absence of the error, i.e., for ϵ=0. This error takes account of the potential imprecise or incomplete knowledge of both the actual coupling between the two levels, on the one hand, and of the exact level spacing at the beginning and at the end of the sweep. These types of errors are common, for instance, in experiments controlling single cold-atom collisions [41,42,43]. To track the impact of such an error on the performance of the counterdiabatic protocols, the system is initially prepared in state $|0\rangle \dot{=}\left(\begin{array}{c} 0\\ 1 \end{array}\right)$, and the final fidelity $F_{1}$, i.e., the probability of being in the target state $|1\rangle \dot{=}\left(\begin{array}{c} 1\\ 0 \end{array}\right)$,

(11) $$F_{1}={\left|\;\langle \psi (\tau =1)|1\rangle \;\right|}^{2},$$

is monitored as a function of the total time evolution and ϵ. The results are shown in Figure 2. As evident from that figure, all protocols are not very sensitive to the error and confirm the effectiveness of STA protocols for control in a closed-system setting.

In the open system scenario, instead, we consider a single decay channel whose jump operator $\hat{c}$ is a superposition of the two standard operators ${\hat{\sigma }}_{x}$ and ${\hat{\sigma }}_{z}$, typically used in quantum information theory [44]

(12) $${\hat{c}}_{\theta }=\sqrt{\gamma }\left[\cos\left(\theta \right){\hat{\sigma }}_{z}+\sin\left(\theta \right){\hat{\sigma }}_{x}\right]\;,$$

where γ is measured in units of $1/t_{f}$. The effect of this operator can be understood by considering two limiting cases. In the limit θ=0, this operator describes pure dephasing—that is, the populations of the density matrix are preserved, while off-diagonal elements, i.e., the coherences, decay to zero. This type of noise may result from an off-resonant interaction with the environment in which energy exchange, and thus absorption/emission, is strongly unfavored and may also describe the backaction resulting from a quantum nondemolition measurement of the system state [40,45,46]. For θ=π/2, the decay of coherences accompanies population relaxation, leading to a steady state, which can be viewed as an infinite temperature state, represented by a diagonal density matrix with equal entries 1/2. Intermediate values of θ interpolate the two limiting situations, essentially controlling the rate of population relaxation as compared to the dephasing times. In the following, we considered γ values with upper bounds given by two specific experimental realization: cold Rydberg atoms [47] and superconducting qubits [48] with maximal γ∝0.1 and ∝1 in our units, respectively.

**Figure 3:**
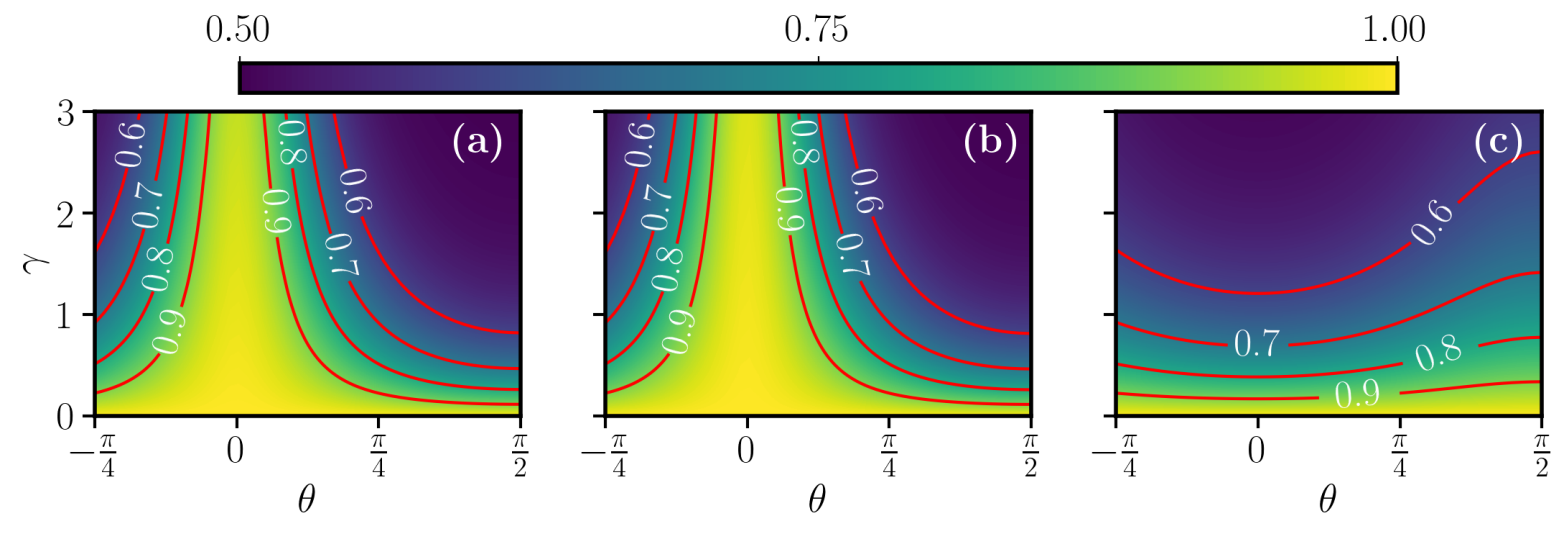
Color map of the final fidelity of Equation (11) at the end of the control protocols for the STA corresponding to $f_{L}\left(\tau \right)$ in (**a**), $f_{\mathrm{PL}}\left(\tau \right)$ in (**b**), $f_{\mathrm{RC}}\left(\tau \right)$ in (**c**) for the jump operator of the Equation (12) as a function of θ and of the decay rate γ. The first two control protocol are very stable around θ=0 and also in this case, (**a**,**b**) look very similar for the same reason explained in Figure 2. Panel (**c**), instead, presents the opposite behavior, with respect to (**a**,**b**), being more robust around θ=π/2. The other simulation parameters are the same as in Figure 1.

The final fidelity as a function of the angle θ for different sweep functions is depicted in Figure 3. The different counterdiabatic protocols now behave rather differently. One can notice that the counterdiabatic protocol for $H\{f_{L}\}$, corresponding to a linear ramp, gives best fidelities for θ close to zero, namely in the limit of pure dephasing, while it loses efficiency very quickly for increasing γ as θ goes to π/2. For a rather large region around θ=0, the fidelity remains above 0.75, with values above 0.9 for γ, which is small compared to the level coupling.

The polynomial-ramp counterdiabatic protocol for $H\{f_{\mathrm{PL}}\}$ exhibits qualitatively the same behavior but is able to reach much larger fidelities. Remarkably, the fidelity remains close to one in the vicinity of θ=0, even for rather large values of γ. This insensitivity to the pure $\sigma_{z}$ dephasing (diagonal noise), is explained by the fact that the time scale of the population transfer given by the linear and the polynomial sweep functions is much faster than the time scale of the noise 1/γ. In contrast, the Roland–Cerf is very slow around the avoided crossing, of the order 1/γ. The counterdiabatic protocol for $H\{f_{\mathrm{RC}}\}$ associated to the Roland–Cerf sweep function shows a completely different behavior instead. This protocol gives poor performance when subject to pure dephasing, whilst it is more robust against transverse noise, i.e., for θ close to π/2. Despite this robustness, the best fidelities reached in this case for large γ are still rather small as compared to the target value $F_{1}=1$.

**Figure 4:**
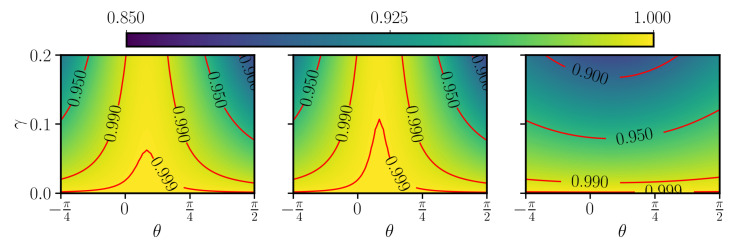
Zoom into the high fidelity region ($F_{1}>0.85$) for the data in Figure 3. We see that the hierarchy established in Figure 3 between the three sweep functions is maintained.

The observed behavior of the different counterdiabatic protocols with respect to the different types of noise described by the varying parameter θ can be explained as follows, by inspecting the adiabatic states of the system. Let us first recall that, under counterdiabatic driving, the isolated system perfectly follows the instantaneous eigenstates of the Hamiltonian $H_{0}\left(t\right)$. For the polynomial sweep function $f_{\mathrm{PL}}\left(t\right)$, these eigenstates remain for most of the time evolution close to an eigenstate of ${\hat{\sigma }}_{z}$, departing significantly from initial |0〉 only for a brief interval at the avoided crossing, when the population transfer abruptly occurs, before the population settles in the |1〉 eigenstate. This can be observed in Figure 1c. Thus, in the limit of pure dephasing (θ=0), the system is for the largest part of the evolution in an eigenstate of the jump operator $\hat{c}≃{\hat{\sigma }}_{z}$ and, as such, is insensitive to the interaction with the environment. To confirm this interpretation, one can roughly estimate the loss in fidelity by coarse-graining the dynamics into two regimes, similarly to what was done in the adiabatic-impulse approximation [49]. In a first regime, which describes the evolution before and after the avoided crossing, we assume that the state is exactly in |0〉 or |1〉 and no fidelity is lost. In a second regime, the state is a superposition of |0〉 or |1〉 and the decay induces errors. Differently from $f_{L}$ and $f_{\mathrm{PL}}$, the function $f_{\mathrm{RC}}$ quickly brings the two levels close to each other and then slows down the evolution in the vicinity of the avoided crossing. Then, the system is for most of the evolution in a superposition state a|0〉+b|1〉 and is thus much more prone to dephasing. We conclude that, while in a closed-system scenario all the different protocols considered essentially give very similar performance, the introduction of incoherent processes discriminates different protocols. From Figure 3, one can clearly conclude that the counterdiabatic method of choice can strongly depend on the specifics of the incoherent noise acting on the system. For a quantum computing purpose, where high fidelity is required, in Figure 4, we show a restricted region of Figure 3 with high fidelity ($F_{1}>0.85$). We notice that the hierarchy between the three control protocols, established from Figure 3, holds also in the high-fidelity region.

The second type of noise considered is the one given by a thermal bath at zero temperature, describing, for instance, spontaneous emission of a two-level atom. This is modeled by the master equation in Equation (7) with dissipator $\hat{c}=\sqrt{\gamma_{-}}\sigma_{-}$, where $\sigma_{-}$ is the spin-lowering operator.

**Figure 5:**
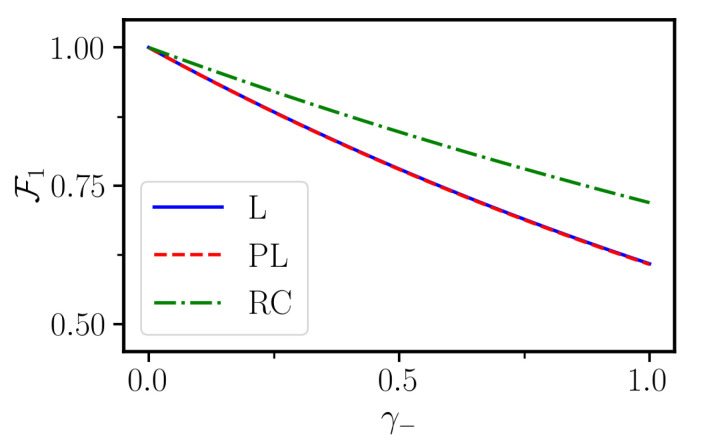
Final fidelity at the end of the different STA control protocol as a function of the decay rate $\gamma_{-}$ for a jump operator ${\hat{\sigma }}_{-}$, of the three sweep functions linear (blue solid line), polynomial (dashed red line) and Roland–Cerf (green dashed-dotted line). It can be seen that the linear and the polynomial control protocol have the same behavior because of the very similar occupation time of the state |1〉. On the contrary, with the Roland–Cerf, the population of state |1〉 is occupied later than the other two, and therefore it is more robust. The other simulation parameters are the same as in Figure 1.

The final fidelity at the end of the protocol is depicted in Figure 5 as a function of the spontaneous emission rate $\gamma_{-}$.

The linear and polynomial sweep functions give almost indistinguishable results and the corresponding curves in Figure 5 are essentially overlapping. The Roland–Cerf function gives instead larger fidelity for all values of $\gamma_{-}$. This can be understood by noting that the decay becomes active when state |1〉 becomes populated. Then, one can see from Figure 1c that such a state gets populated much more quickly when using the linear or polynomial ramp. Hence, these protocols are more vulnerable to decay than the Roland–Cerf protocol, which instead populates state |1〉 more slowly.

### 3.2. Two-Qubit Gate

The second quantum system we consider is given by two interacting qubits with the following Hamiltonian:

(13) $$H_{2q}\left(t\right)=\frac{\omega_{1}}{2}{\hat{\sigma }}_{1}^{z}+\left(\frac{\omega_{1}}{2}+\alpha f\left(t\right)\right){\hat{\sigma }}_{2}^{z}+g\left({\hat{\sigma }}_{1}^{+}⊗{\hat{\sigma }}_{2}^{-}+{\hat{\sigma }}_{1}^{-}⊗{\hat{\sigma }}_{2}^{+}\right)\;,$$

where $\left\{{\hat{\sigma }}_{k}^{x},{\hat{\sigma }}_{k}^{y},{\hat{\sigma }}_{k}^{z}\right\}$ and $\left\{{\hat{\sigma }}_{k}^{\pm }=\frac{1}{2}\left[{\hat{\sigma }}_{k}^{x}\pm i{\hat{\sigma }}_{k}^{y}\right]\right\}$ are the Pauli matrices and the raising and lowering operators of the *k*-th qubit (k=1,2), respectively, and $\omega_{1}$ is the transition frequency associated to the qubit levels. The interaction describes an energy-conserving exchange of excitations between the qubits, which can be exploited to realize an entangling gate. Indeed, while this interaction does not involve the two-qubit states |00〉 and |11〉, it implements a rotation in the subspace spanned by states |01〉 and |10〉, which can thus produce the entangled Bell states $|{\mathrm{Bell}}_{\pm }\rangle =\frac{1}{\sqrt{2}}\left[|01\rangle \pm |10\rangle \right]$. Therefore, starting from the initial state |01〉, in this case the fidelity is evaluated with respect to the state $|{\mathrm{Bell}}_{+}\rangle$ as follows:

(14) $$F_{2}={\left|\langle \psi \left(\tau =1\right)|{\mathrm{Bell}}_{+}\rangle \right|}^{2}\;.$$

**Figure 6:**
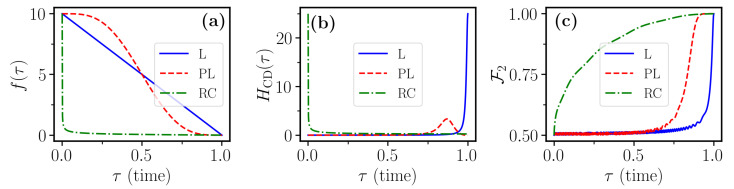
Temporal profile for the two-qubit case of (**a**) the same three sweep functions of Section 2.1; (**b**) the coefficients of the matrix elements in Equation (16) of the counterdiabatic term, for the three sweep functions; (**c**) the fidelity $F_{2}$ of Equation (14), in the absence of any error and dissipation (γ=0). The simulation parameters are $\omega_{g1}=30,T=10,\alpha_{g}=10,f_{0}=10,\gamma =0$.

This system has been studied in the context of closed-systems STA for the specific realization by two-coupled superconducting qubits [35]. In this specific implementation [48,50,51,52], the interaction of the form given in Equation (13) may arise from a direct capacitive coupling of the superconducting qubits, or when the qubits are dispersively coupled to a cavity, being mediated by a cavity through the exchange of virtual photons [50,53,54]. The sweep function f(τ) describes a modulation of the detuning between the two qubits, and in Figure 6a the temporal dependence of the sweep functions that we chose is shown. The corresponding counterdiabatic corrections $H_{\mathrm{CD}}\left(\tau \right)$ are depicted in Figure 6b, which ensure the perfect following of the instantaneous eigenstate time evolution. When f(τ) is large, the qubits are off-resonant and do not exchange excitations. As f(τ) goes to zero, the interaction becomes resonant and the states |01〉 and |10〉 enter an avoided crossing of width 2g. The eigenstates of the system are thus approximately factorized when f(τ) is far from zero, while two eigenstates are entangled for f(τ)=0. The adiabatic entangling protocol thus consists, in this case, in slowly sweeping f(τ) from a large value to zero. Using the same parametrization for the two-level scheme—see discussion before Equation (9)—also in this case, we rewrite the Hamiltonian in natural units, leading to the following:

(15) $$H_{2q}\left(\tau \right)=T\left[\frac{\omega_{g1}}{2}{\hat{\sigma }}_{1}^{z}+\left[\frac{\omega_{g1}}{2}+\alpha_{g}f\left(\tau \right)\right]{\hat{\sigma }}_{2}^{z}+\left[{\hat{\sigma }}_{1}^{+}⊗{\hat{\sigma }}_{2}^{-}+{\hat{\sigma }}_{1}^{-}⊗{\hat{\sigma }}_{2}^{+}\right]\right]\;.$$

The counterdiabatic Hamiltonian for a generic sweep function f(τ) can be derived analytically and reads as follows:

(16) $$H_{\mathrm{CD}}\left(\tau \right)=\frac{\alpha_{g}\dot{f}\left(\tau \right)}{4[1+f^{2}\left(\tau \right)]}\left(\sigma_{1}^{y}⊗\sigma_{2}^{x}-\sigma_{1}^{x}⊗\sigma_{2}^{y}\right)\;.$$

The field in Equation (16) is in general difficult to realize experimentally, but it can be realized using approximate schemes as proposed, for example, in refs. [18,35]. In the absence of noise, the time evolution of the counterdiabatic terms and the corresponding fidelities are shown in Figure 6b,c, respectively.

**Figure 7:**
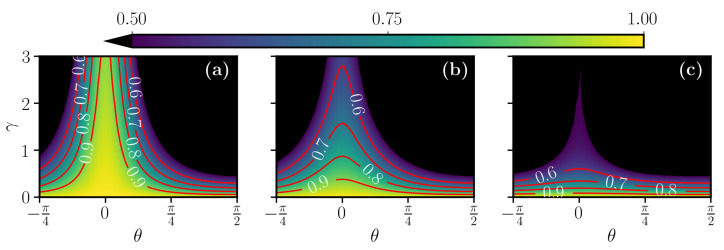
Final fidelity of Equation (14) at the end of the different STA control protocols, (**a**) linear, (**b**) polynomial, and (**c**) Roland–Cerf, as a function of θ and the common rate γ. In contrast with the one-qubit case, the linear and the polynomial protocols behave differently because of their different temporal profile of the fidelity, as shown in Figure 6c. In this case, the linear is more robust than the other two around θ=0. The other simulation parameters are the same as in Figure 6.

We then study how the presence of dissipative effects reduces the performance of the control protocol. As a start, the qubits are assumed to be subject to the same type of noise, namely, they couple with equal strength γ to the environment through a jump operator as in Equation (12). Following Equation (7), the master equation for our two qubits system now reads as follows:

(17) $$\frac{d\rho (\tau )}{d\tau }=-i\left[H_{2q}\left\{f\left(\tau \right)\right\},\rho \left(\tau \right)\right]+D\left[{\hat{c}}_{1}\left(\theta \right)\right]\rho \left(\tau \right)+D\left[{\hat{c}}_{2}\left(\theta \right)\right]\rho \left(\tau \right),$$

with ℏ=1 and ${\hat{c}}_{k}\left(\theta \right)=\sqrt{\gamma }\left[\cos\left(\theta \right){\hat{\sigma }}_{k}^{z}+\sin\left(\theta \right){\hat{\sigma }}_{k}^{x}\right]$. The final fidelity as a function of θ and γ is shown in Figure 7. For this type of transfer control, the linear sweep function produces a steeper state transfer than the other two as we can deduce from Figure 6c, which means that the time during which the system is in a superposition of states is very short, and in fact it is more robust against pure dephasing ${\hat{c}}_{k}≃\sigma_{k}^{z}$. On the contrary, with the Roland–Cerf protocol, the system is for a longer time in a superposition of the participating states |10〉 and |01〉, and in fact it is less robust with respect to the others. Regarding the decoherence generated by the jump operator ${\hat{c}}_{k}≃\sigma_{k}^{x}$, all the three sweep functions behave in the same way. None of the protocols shows a resistance against such a decoherence channel when acting equally on both the atoms. In fact, the eigenstates of the system do not tend to the eigenstate of the collapse operator, as we have already seen in the one-atom case.

**Figure 8:**
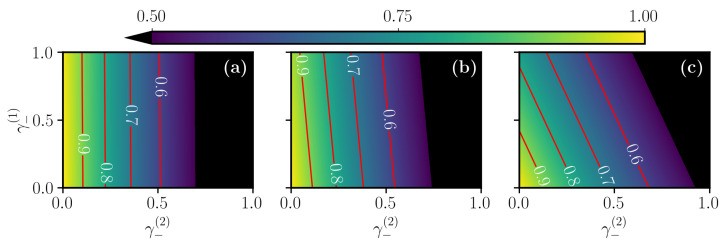
Final fidelity at the end of the different STA protocols, (**a**) linear, (**b**) polynomial and (**c**) Roland–Cerf, as a function of the two different local decay rates of the two qubits. The other simulation parameters are the same as in Figure 6.

The effect of spontaneous emissions, described by the jump operators ${\hat{c}}_{k}=\sqrt{\gamma_{-}}\;\sigma_{k}^{-}$, on our two-qubit system that we consider is shown in Figure 8. In this picture, we compare the effect of different decay rates acting on the two atoms, still for the same three sweep functions. To explain what we see, we recall that the initial state is |01〉 while the target state is $|{\mathrm{Bell}}_{+}\rangle =\frac{1}{\sqrt{2}}\left(|10\rangle +|01\rangle \right)$. We see from Figure 8 that the first qubit becomes more sensitive as the protocol changes from Figure 8a to Figure 8c. In fact, the first qubit which starts from the state |0〉 is less sensitive to the decay when the sweep function is initially very slow and then fast toward the end. As a consequence, the population of the level |1〉 is low for a very long time such that the first atom is more insensitive to the decay as shown in Figure 8a. The polynomial protocol in Figure 8b is also quite robust but slightly less than the linear one. The reason for this can be appreciated from Figure 6c, where we see that the polynomial sweep populates the state |1〉 of the first qubit shortly before the linear one. On the contrary, in the Roland–Cerf protocol, the population of the state |1〉 of the first atom starts to be populated early in time, and this is the reason why it suffers more the effect of the decay by $\gamma_{-}^{\left(1\right)}.$ For the second atom, instead, which starts from the state |1〉, the situation is the opposite. In fact, with the Roland–Cerf protocol, the state |1〉 loses population before the other two sweep functions, giving a little more robustness against decay to the second atom as indicated by the tilted contour lines in Figure 8c. Moreover, looking at the diagonals in Figure 8, we observe very similar behavior when the two systems are subject to the same decay rate $\gamma_{-}^{\left(1\right)}=\gamma_{-}^{\left(2\right)}$.

## 4. Discussion and Conclusions

In this work, the performance of control protocols exploiting different shortcut-to-adiabatic evolutions was investigated in the presence static imperfections, and dissipation and dephasing. In particular, we first studied for the one-qubit case the robustness of the STA protocols against static uncertainties in the uncoupled energy gap, against diagonal and off-diagonal dephasing, and finally against spontaneous emission. Then, we investigated two coupled qubits experiencing the same local dephasing or subject to individual spontaneous emission. The results show that, while all STA are fairly robust against parameter imperfections in the control fields, they give substantially different performance when the interaction of the system with the environment is included in the picture. Dephasing and dissipation strongly discriminate the different protocols, making some less efficient than others.

Our findings indicate a clear path toward the design of robust control protocols for open quantum systems. This is based on the idea of engineering the rate of the adiabatic following, which is enforced by the shortcut-to-adiabaticity fields, such as minimizing the sensitivity of the system to the external perturbation. The latter goal can be achieved, on the one hand, by minimizing the fraction of the evolution that the system spends in particularly decay-prone state and, on the other hand, by maximizing the time spent in states particularly insensitive to the dissipative and incoherent processes. The latter states are close to a stationary state of the dissipator in the master equation, namely, close to a state $\rho_{\psi }=\left|\psi \rangle \langle \psi \right|$ such that $D\left[\hat{c}\right]\rho_{\psi }=0$ for a given jump operator $\hat{c}$.

## Data Availability

Data can be provided upon reasonable request.

## Abbreviations

The following abbreviations are used in this manuscript:

STA	shortcuts to adiabaticity
CD	counterdiabatic
L	linear
PL	polynomial
RC	Roland–Cerf

## Appendix A. Roland–Cerf Sweep Function

In this appendix, following the steps already presented in [34,35,55], we review how to adapt the Roland–Cerf (RC) function for controlling the evolution in our two-level system in Section 3.1. This exemplary case illustrates the practically useful procedure for computing the sweeps for more complex systems. For the purpose, let us start from a Hamiltonian of the following form:

(A1) $$H\left(t\right)=\alpha f\left(t\right)\sigma_{z}+g\sigma_{x}\;,$$

where α>0 and *g* are the same parameters defined in the text, before Equation (9). Let us consider the gap between the eigenenergies of systems $E_{+}$ and $E_{-}$, corresponding to the excited $|\psi_{+}\left[f\left(t\right)\right]\rangle$ and ground states $|\psi_{-}\left[f\left(t\right)\right]\rangle$, respectively:

(A2) $$\Delta E\left[f\left(t\right)\right]=E_{+}-E-=2\alpha \sqrt{f^{2}\left(t\right)+{\left(\frac{g}{\alpha }\right)}^{2}}\;.$$

Now, after finding the expression of the two eigenstates and computing $\partial_{f}H$, we can impose the local adiabatic condition as follows [34,56]:

(A3) $$\left|\frac{df}{dt}\right|\frac{\left|\langle e\left(f\right)|\partial_{f}H|g\left(f\right)\rangle \right|}{{\left(\Delta E\right)}^{2}}<\epsilon \;.$$

Demanding also that f(t) is monotonically decreasing $\frac{df}{dt}\leq 0$, as in Figure 1a and Figure 6a, this leads to the following:

(A4) $$\frac{dt}{df}=-\frac{\left|g\right|}{4\epsilon \alpha^{2}{\left[{\left(\frac{g}{\alpha }\right)}^{2}+f^{2}\left(t\right)\right]}^{3/2}}\;,$$

where we consider the threshold value from Equation (A3). For convenience, we first integrate Equation (A4) between $f\left(0\right)=f_{0}>0$ and $f\left(t_{f}\right)=-f_{0}$, obtaining the expression for the final time as follows:

(A5) $$t_{f}=\frac{f_{0}}{2\epsilon |g|\sqrt{{\left(\frac{g}{\alpha }\right)}^{2}+f_{0}^{2}}}\;.$$

Having the final time $t_{f}$, we can now find f(t) by integrating again Equation (A4), this time from f(0) to f(t), and then by inverting the resulting function t(f). The RC sweep function then reads as follows:

(A6) $$f\left(t\right)=\frac{\left(\frac{t_{f}}{2}-t\right)4\epsilon {\left|g\right|}^{2}}{\alpha \sqrt{1-{\left(\frac{t_{f}}{2}-t\right)}^{2}16\epsilon^{2}{\left|g\right|}^{2}}}\;.$$

Finally, in order to obtain exactly Equation (4), we just introduce the dimensionless time $\tau =t/t_{f}$ and $\alpha_{g}=\alpha /g$; rearranging, the RC sweep function now takes the following form:

(A7) $$f_{RC}\left(\tau \right)=\frac{\left(1-2\tau \right)f_{0}}{\sqrt{1+4\alpha_{g}^{2}f_{0}^{2}\tau \left(1-\tau \right)}}\;.$$

The RC sweep function used in Section 3.2 is computed numerically along similar lines.

## References

[1] Vitanov N.V., Rangelov A.A., Shore B.W., Bergmann K. (2017). Stimulated Raman adiabatic passage in physics, chemistry, and beyond. Rev. Mod. Phys..

[2] Král P., Thanopulos I., Shapiro M. (2007). Colloquium: Coherently controlled adiabatic passage. Rev. Mod. Phys..

[3] Messiah A. (1961). Quantum Mechanics.

[4] Rezakhani A.T., Kuo W.J., Hamma A., Lidar D.A., Zanardi P. (2009). Quantum Adiabatic Brachistochrone. Phys. Rev. Lett..

[5] Lidar D.A., Rezakhani A.T., Hamma A. (2009). Adiabatic approximation with exponential accuracy for many-body systems and quantum computation. J. Math. Phys..

[6] Albash T., Lidar D.A. (2015). Decoherence in adiabatic quantum computation. Phys. Rev. A.

[7] Mohseni N., Narozniak M., Pyrkov A.N., Ivannikov V., Dowling J.P., Byrnes T. (2021). Error suppression in adiabatic quantum computing with qubit ensembles. NPJ Quantum Inf..

[8] Guéry-Odelin D., Ruschhaupt A., Kiely A., Torrontegui E., Martínez-Garaot S., Muga J.G. (2019). Shortcuts to adiabaticity: Concepts, methods, and applications. Rev. Mod. Phys..

[9] Torrontegui E., Ibáñez S., Martí nez-Garaot S., Modugno M., del Campo A., Guéry-Odelin D., Ruschhaupt A., Chen X., Muga J.G. (2013). Shortcuts to Adiabaticity. Adv. At. Mol. Opt. Phys..

[10] del Campo A., Kim K. (2019). Focus on Shortcuts to Adiabaticity. New J. Phys..

[11] Ibáñez S., Chen X., Torrontegui E., Muga J.G., Ruschhaupt A. (2012). Multiple Schrödinger Pictures and Dynamics in Shortcuts to Adiabaticity. Phys. Rev. Lett..

[12] del Campo A. (2013). Shortcuts to Adiabaticity by Counterdiabatic Driving. Phys. Rev. Lett..

[13] Baksic A., Ribeiro H., Clerk A.A. (2016). Speeding up Adiabatic Quantum State Transfer by Using Dressed States. Phys. Rev. Lett..

[14] Martínez-Garaot S., Torrontegui E., Chen X., Muga J.G. (2014). Shortcuts to adiabaticity in three-level systems using Lie transforms. Phys. Rev. A.

[15] Chen X., Torrontegui E., Muga J.G. (2011). Lewis-Riesenfeld invariants and transitionless quantum driving. Phys. Rev. A.

[16] Petiziol F., Dive B., Mintert F., Wimberger S. (2018). Fast adiabatic evolution by oscillating initial Hamiltonians. Phys. Rev. A.

[17] Sels D., Polkovnikov A. (2017). Minimizing irreversible losses in quantum systems by local counterdiabatic driving. Proc. Natl. Acad. Sci. USA.

[18] Opatrný T., Mølmer K. (2014). Partial suppression of nonadiabatic transitions. New J. Phys..

[19] Chen Y.H., Xia Y., Wu Q.C., Huang B.H., Song J. (2016). Method for constructing shortcuts to adiabaticity by a substitute of counterdiabatic driving terms. Phys. Rev. A.

[20] Giannelli L., Arimondo E. (2014). Three-level superadiabatic quantum driving. Phys. Rev. A.

[21] Eckardt A. (2017). Colloquium: Atomic quantum gases in periodically driven optical lattices. Rev. Mod. Phys..

[22] Decker K.S.C., Karrasch C., Eisert J., Kennes D.M. (2020). Floquet Engineering Topological Many-Body Localized Systems. Phys. Rev. Lett..

[23] Claeys P.W., Pandey M., Sels D., Polkovnikov A. (2019). Floquet-Engineering Counterdiabatic Protocols in Quantum Many-Body Systems. Phys. Rev. Lett..

[24] Sameti M., Hartmann M.J. (2019). Floquet engineering in superconducting circuits: From arbitrary spin-spin interactions to the Kitaev honeycomb model. Phys. Rev. A.

[25] Bukov M., D’Alessio L., Polkovnikov A. (2015). Universal high-frequency behavior of periodically driven systems: From dynamical stabilization to Floquet engineering. Adv. Phys..

[26] Boyers E., Pandey M., Campbell D.K., Polkovnikov A., Sels D., Sushkov A.O. (2019). Floquet-engineered quantum state manipulation in a noisy qubit. Phys. Rev. A.

[27] Villazon T., Claeys P.W., Polkovnikov A., Chandran A. (2021). Shortcuts to dynamic polarization. Phys. Rev. B.

[28] Bartels B., Mintert F. (2013). Smooth optimal control with Floquet theory. Phys. Rev. A.

[29] Lucas F., Mintert F., Buchleitner A. (2013). Tailoring many-body entanglement through local control. Phys. Rev. A.

[30] Zhou B.B., Baksic A., Ribeiro H., Yale C.G., Heremans F.J., Jerger P., Auer A., Burkard G., Clerk A.A., Awschalom D.D. (2017). Accelerated quantum control using superadiabatic dynamics in a solid-state lambda system. Nat. Phys..

[31] Masuda S., Rice S.A. (2015). A model study of assisted adiabatic transfer of population in the presence of collisional dephasing. J. Chem. Phys..

[32] Levy A., Torrontegui E., Kosloff R. (2017). Action-noise-assisted quantum control. Phys. Rev. A.

[33] Levy A., Kiely A., Muga J.G., Kosloff R., Torrontegui E. (2018). Noise resistant quantum control using dynamical invariants. New J. Phys..

[34] Roland J., Cerf N.J. (2002). Quantum search by local adiabatic evolution. Phys. Rev. A.

[35] Petiziol F., Dive B., Carretta S., Mannella R., Mintert F., Wimberger S. (2019). Accelerating adiabatic protocols for entangling two qubits in circuit QED. Phys. Rev. A.

[36] Berry M.V. (2009). Transitionless quantum driving. J. Phys. A.

[37] Demirplak M., Rice S.A. (2003). Adiabatic Population Transfer with Control Fields. J. Phys. Chem. A.

[38] Breuer H.P., Petruccione F. (2007). The Theory of Open Quantum Systems.

[39] Berry M.V. (1995). Two-State Quantum Asymptotics. Ann. N. Y. Acad. Sci..

[40] Walls D.F., Milburn G.J. (2008). Quantum Optics.

[41] Sompet P., Szigeti S.S., Schwartz E., Bradley A.S., Andersen M.F. (2019). Thermally robust spin correlations between two 85Rb atoms in an optical microtrap. Nat. Commun..

[42] Reynolds L.A., Schwartz E., Ebling U., Weyland M., Brand J., Andersen M.F. (2020). Direct Measurements of Collisional Dynamics in Cold Atom Triads. Phys. Rev. Lett..

[43] Weyland M., Szigeti S.S., Hobbs R.A.B., Ruksasakchai P., Sanchez L., Andersen M.F. (2021). Pair Correlations and Photoassociation Dynamics of Two Atoms in an Optical Tweezer. Phys. Rev. Lett..

[44] Nielsen M.A., Chuang I.L. (2010). Quantum Computation and Quantum Information.

[45] Clerk A.A., Devoret M.H., Girvin S.M., Marquardt F., Schoelkopf R.J. (2010). Introduction to quantum noise, measurement, and amplification. Rev. Mod. Phys..

[46] Ralph T.C., Bartlett S.D., O’Brien J.L., Pryde G.J., Wiseman H.M. (2006). Quantum nondemolition measurements for quantum information. Phys. Rev. A.

[47] Zhang C., Pokorny F., Li W., Higgins G., Pöschl A., Lesanovsky I., Hennrich M. (2020). Submicrosecond entangling gate between trapped ions via Rydberg interaction. Nature.

[48] Blais A., Grimsmo A.L., Girvin S.M., Wallraff A. (2021). Circuit quantum electrodynamics. Rev. Mod. Phys..

[49] Damski B., Zurek W.H. (2006). Adiabatic-impulse approximation for avoided level crossings: From phase-transition dynamics to Landau-Zener evolutions and back again. Phys. Rev. A.

[50] Blais A., Huang R.S., Wallraff A., Girvin S.M., Schoelkopf R.J. (2004). Cavity quantum electrodynamics for superconducting electrical circuits: An architecture for quantum computation. Phys. Rev. A.

[51] Krantz P., Kjaergaard M., Yan F., Orlando T.P., Gustavsson S., Oliver W.D. (2019). A quantum engineer’s guide to superconducting qubits. Appl. Phys. Rev..

[52] Gu X., Kockum A.F., Miranowicz A., Liu Y., Nori F. (2017). Microwave photonics with superconducting quantum circuits. Phys. Rep..

[53] Blais A., Gambetta J., Wallraff A., Schuster D.I., Girvin S.M., Devoret M.H., Schoelkopf R.J. (2007). Quantum-information processing with circuit quantum electrodynamics. Phys. Rev. A.

[54] Majer J., Chow J.M., Gambetta J.M., Koch J., Johnson B.R., Schreier J.A., Frunzio L., Schuster D.I., Houck A.A., Wallraff A. (2007). Coupling superconducting qubits via a cavity bus. Nature.

[55] Bason M.G., Viteau M., Malossi N., Huillery P., Arimondo E., Ciampini D., Fazio R., Giovannetti V., Mannella R., Morsch O. (2012). High-fidelity quantum driving. Nat. Phys..

[56] Amin M.H.S. (2009). Consistency of the Adiabatic Theorem. Phys. Rev. Lett..

